# The Role of Ideation on Intention to Use Contraceptives Among Married Women in Shenen Dhugo District, Eastern Ethiopia

**DOI:** 10.1155/bmri/9361411

**Published:** 2026-09-16

**Authors:** Abrahim Beker, Berhe Gebremichael, Ahmedin Aliyi Usso, Hassen Abdi Adem, Merhawi Gebremedhin

**Affiliations:** ^1^ Gambella General Hospital, Gambella Health Bureau, Gambela, Ethiopia; ^2^ School of Public Health, College of Health and Medical Sciences, Haramaya University, Harar, Ethiopia, haramaya.edu.et; ^3^ School of Nursing and Midwifery, College of Health and Medical Sciences, Haramaya University, Harar, Ethiopia, haramaya.edu.et

**Keywords:** Ethiopia, family planning, ideation, intention to use contraceptives, women

## Abstract

**Background:**

The utilization of modern contraceptives remains low in sub‐Saharan African countries, including Ethiopia. The main barrier to the intention to use contraceptives may be the dimensions of ideation. Therefore, this study assessed the role of ideation on the intention to use contraceptives in Shenen Dhugo district, eastern Ethiopia.

**Methods:**

A community‐based cross‐sectional study was conducted among 501 married women in Shenen Dhugo district, eastern Ethiopia, from September 1 to 30, 2023. Two‐stage stratified sampling techniques were employed to select the participants. The collected data were entered into EpiData Version 3.1 and analyzed using SPSS Version 27.0. Bivariable and multivariable logistic regression were used to analyze the determinants of intention to use contraceptives. An adjusted odds ratio (AOR) with a 95% confidence interval was used to report the strength of the association, and the statistical significance was declared at a *p* − value < 0.05.

**Results:**

In this study, 48.5% (95% CI: 44.1%, 52.9%) of married women intended to use contraceptives. Dimensions of contraceptive ideation: social interaction (AOR = 4.54, 95% CI: 2.73, 7.53), attitude toward contraceptives (AOR = 3.56, 95% CI: 2.11, 6.00), perceived self‐efficacy (AOR = 2.04, 95% CI: 1.45, 3.79), awareness about contraceptives (AOR = 3.76, 95% CI: 1.94, 7.29), and perceived norms (AOR = 2.82, 95% CI: 1.70, 4.69) were found to be the main predictors of contraceptive use intention. Having a higher income (AOR = 3.15, 95% CI: 1.44, 6.90), secondary school education level (AOR = 3.25, 95% CI: 1.20, 8.80), and having used previous contraceptives (AOR = 5.74, 95% CI: 3.27, 10.07) were the factors improving the women′s intention to use contraceptives.

**Conclusion:**

Nearly half of married women intended to use a contraceptive in Shenen Dhugo, eastern Ethiopia. This study also provides evidence about the importance of ideation in helping to promote women′s intention to use contraceptives. Therefore, developing interventions that enhance women′s contraceptive ideation and encouraging couples to determine a preferred family size are innovative strategies for contraceptive self‐efficacy and intention to use. In addition, providing behavioral change health education on contraceptives at every entry point in the health system while addressing existing myths and rumors regarding contraceptive side effects is necessary to strengthen intention and utilization of contraceptives.

## 1. Introduction

Contraceptive use is an innovative strategy that helps to prevent maternal and neonatal morbidity and mortality, as well as improving the lives of women and their families by reducing unintended pregnancy and unsafe abortion [1, 2]. However, the use of modern contraceptives is low in developing countries, which contributes to a higher burden of unintended pregnancy [3]. An unintended pregnancy was more likely to occur among women who did not intend to use contraceptives [4].

An intention to use a modern contraceptive is important to understanding the women′s needs in the future and making them more likely to turn that intention into action. It is the best method of controlling family size, short birth intervals, unintended pregnancies, and induced abortion [5]. Even though the intention to use modern contraceptives is known to improve maternal and child health, the burden of unintended pregnancies, high fertility, and induced abortion was still the main challenge in developing countries [6–8].

Unintended pregnancy is a major risk factor affecting maternal and child health in developing countries. Globally, around 121 million unintended pregnancies occur each year. The burden of unintended pregnancy was 65% in developing countries, 30% in sub‐Saharan Africa (SSA) countries, and 38% in Ethiopia. This higher burden was associated with lower use of modern contraceptives [3, 9–11].

The level of modern contraceptive use among married women was 58% [12]. According to the Ethiopia Demographic and Health Survey (EDHS) 2019, the national modern contraceptive utilization was 40.5%, which is lower in pastoral regions: 12.7% in Afar and 3.4% in Somali [13]. This low utilization of modern contraceptives is associated with women′s low intention to use modern contraceptives [14].

The proportion of intention to use modern contraceptives is higher in developed countries, with a rate of 91% in the United States, whereas it is lower in Eastern Africa, including Ethiopia. The intention to use modern contraceptives in different parts of Ethiopia was 18.2% in Oromia, 28% in Benishangul‐Gumuz, 35% in Gambela, 12% in Afar, and 1% in Somali [9, 15, 16].

Multiple risk factors affect women′s intention to use modern contraceptives, such as the socioeconomic status of the women, residential area, and parity [17]. The previous study revealed that the intention to use modern contraceptives is influenced by factors such as gender preference, family size, maternal age, and maternal educational status [18, 19].

Despite family planning and other fertility regulation programs being implemented in many low‐ and middle‐income countries (LMICs) to give women the opportunity to control childbearing, the proportion of women who intended to use contraceptives remained low [20]. Intention to use contraceptives is a key predictor of future contraceptive behaviors and reflects motivational and cognitive readiness to use contraceptives [21]. In addition, the Ethiopian government committed significant resources to implement a reproductive health program that encourages the use of high‐quality contraceptive services to increase the intention and use of contraceptives among married women. However, the intention to use contraceptives remained low in Ethiopia [22]. Therefore, it is important to address the vital barriers to intention to use contraceptives and to design an appropriate strategy to improve the intention and utilization of services.

Ideation is the concept that an individual′s actions are influenced by their beliefs, ideas, and feelings. It provides a framework for understanding contraceptive behavior. The ideation model conceptualizes and structures the psychosocial factors of behavior that enable a greater understanding of multiple factors that determine intention and utilization of contraceptive behaviors [23] . Despite its importance in understanding contraceptive behaviors, evidence is scarce regarding the role of ideation in intention to use contraceptives in the country, particularly the eastern part of Ethiopia. Therefore, this study is aimed at assessing the role of ideation on intention to use contraceptives among married women in the reproductive age group in Shenen Dhugo district, eastern Ethiopia.

## 2. Materials and Methods

### 2.1. Study Design, Setting, and Period

A community‐based cross‐sectional study was conducted among married women living in Shenen Dhugo district, eastern Ethiopia, from September 1 to 30, 2023. Shenen Dhugo is one of 15 districts of the west Hararghe zone, located 395 km east of Addis Ababa (the capital city of Ethiopia) and 69 km east of Chiro (the capital town of the west Hararghe zone). Administratively, the district has 26 kebeles (25 rural and one urban) with an estimated total population of 215,976, with 105,829 females, 47,515 women in the reproductive age group, and 21,000 married women in the district. According to the district health office report of 2022, there are 26 health posts, seven health centers, and one nongovernmental organization, Family Guidance Association of Ethiopia (FGAE).

### 2.2. Population

All currently married women who were living in Shenen Dhugo district were the source of the population. All currently married women who were living in the randomly selected kebeles of Shenen Dhugo district during the study period were the study population. Currently married women who were permanently living in the district and those who were not using contraceptives were included in the study. The women who were critically ill and unable to respond during the interview were excluded from the study.

### 2.3. Sample Size Determination and Sampling

The sample size (504) was determined by EPI‐Info Version 7.2, using the single population proportion formula based on the following assumptions: 95% confidence interval (CI), 5% margin of error, using 18.2% proportion of intention to use contraceptives of a similar study [15]. Using a design effect of 2 and adding a 10% nonresponse rate, the final calculated sample size was 504.

A two‐stage stratified sampling technique was used to select the study participants. The kebeles were stratified into rural and urban kebeles (25 rural and one urban). Then, nine rural kebeles and one urban kebele were selected by lottery. The calculated sample size was proportionally allocated to each selected kebele based on the number of currently married women in the kebeles. The total number of households with married women was obtained from family folder records at each kebele′s health post. Eligible women were selected using computer‐assisted simple random sampling using the household code number as the sampling frame. When two or more eligible women were found in a single household, one of them was selected by using a lottery method. After informed consent was obtained, a face‐to‐face interview was used to collect the data from selected participants.

### 2.4. Data Collection Tool and Procedure

The data were collected through face‐to‐face interviews using a structured questionnaire adapted from recently published literature [14, 24–27]. A questionnaire first prepared in English was translated into the local language (Afan Oromo) by two who have a good command of both languages and then back to English to check consistency. The questionnaire contains sociodemographic characteristics, reproductive health factors, contraceptive ideation, and intention to use contraceptives. Ten health extension workers collected the data under the supervision of two supervisors and a principal investigator.

Intention to use a contraceptive is assessed using a yes or no question, asking the woman′s intention to use any contraceptive method within the next 12 months. The woman was considered to have an intention to use a contraceptive if she has a plan to use any of the modern contraceptives in the forthcoming 12 months after the interview [28].

Social interaction was measured using 12 items on a 5‐point Likert scale ranging from *never* to *very often*. The responses were dichotomized such that often and very often were coded as “1” (good social interaction) whereas sometimes, rarely, and never were coded as “0” (poor social interaction). Then, the scores for each item were summed to compute a composite index score. The participants who scored at least the mean were categorized as having a good social interaction and having a poor social interaction otherwise [24, 27, 29, 30].

Attitude toward contraceptives was assessed using 15 items of a 5‐point Likert scale ranging from *strongly disagree* to *strongly agree*. The responses were dichotomized such that agree and strongly agree were coded as “1” (positive attitude) whereas neutral, disagree, and strongly disagree were coded as “0” (negative attitude). Then, the scores for each item were summed to compute a composite index score. The participants who scored at least the mean were categorized as having a positive attitude and having a negative attitude otherwise [24, 27, 29, 30].

Self‐efficacy on contraceptive use was assessed using seven items on a 5‐point Likert scale ranging from *strongly disagree* to *strongly agree*. The responses were dichotomized such that agree and strongly agree were coded as “1” (high self‐efficacy) whereas neutral, disagree, and strongly disagree were coded as “0” (low self‐efficacy). Then, the scores for each item were summed to compute a composite index score. The participants who scored at least the mean were categorized as having a high self‐efficacy and having a low self‐efficacy otherwise [24, 27, 29, 30].

Awareness of contraceptives was measured using 12 dichotomized (yes or no) questions about contraceptives. The responses were coded as “1” when they responded yes and coded as “0” when they responded no to the correct questions. Then, the scores for each item were summed to compute a composite index score. The participants who scored at least the mean were categorized as having good contraceptive awareness and having poor contraceptive awareness otherwise [24, 27, 29, 30].

Perceived norms was assessed using 12 items on a 5‐point Likert scale, with response options ranging from *strongly agree* to *strongly disagree* for negatively worded statements. The responses were dichotomized such that disagree and strongly disagree were coded as “1” (perceived right) whereas neutral, agree, and strongly agree were coded as “0” (perceived wrong). Then, the scores for each item were summed to compute a composite index score. The participants who scored at least the mean were categorized as having perceived right norms about contraceptives and having perceived wrong norms about contraceptives otherwise [29, 31].

### 2.5. Data Quality Control

The questionnaires prepared in English were translated into the local language (Afan Oromo) and back to English by two experts with a good command of both languages. We pretested the adapted questionnaire on 5% of the sample size (25 participants) to check validity in a separate kebele in the district before the actual data collection. The data collectors and supervisors were trained for one day on the objectives of the study and the data collection technique.

### 2.6. Data Processing and Analysis

After confirming the completeness of the collected data, the data were entered into EpiData Version 3.1 and then exported to SPSS Version 27 for analysis. Descriptive statistics, including frequency, measures of central tendency, and dispersion, were used to characterize the participants. Internal consistency was estimated for each dimension′s items using Cronbach′s alpha. Internal consistency was confirmed with Cronbach′s alpha scores of 0.932, 0.828, 0.827, 0.722, and 0.752 for social interaction, attitude, perceived self‐efficacy, awareness, and perceived norms, respectively.

The bivariable and multivariable logistic regression analyses were conducted to identify factors associated with the intention to use a contraceptive. A multivariable logistic regression model was fitted to identify significant risk factors. Hosmer and Lemeshow′s goodness‐of‐fit test with a chi‐square value of 7.081, degree of freedom 8, and *p*‐value of 0.528 was used to confirm the overall adequacy of the model′s fitness. An adjusted odds ratio (AOR) with 95% CI was used to report the strength of an association and the statistical significance, declared at *p* − value < 0.05.

### 2.7. Ethical Approval

The study was conducted following the Helsinki Declaration of research involving human subjects [32]. The study was also approved by the Institutional Health Research Ethical Review Committee of the College of Health and Medical Sciences, Haramaya University, Ethiopia (Ref. No: IHRERC/109/2022). Written informed consent was obtained from all participants after explaining the purpose and benefits of the study.

## 3. Results

### 3.1. Sociodemographic Characteristics

A total of 501 women were enrolled, representing a 99.4% response rate. The mean (± SD) age of the women was 30 (± 5) years, and more than half (55.1%) of the participants were in the age group of 25–34 years. The majority (47.5%) of the study participants had a secondary school education. The majority (42.5%) of husbands had a college or higher education. Regarding occupation status, the majority (87.0%) of the participants were housewives, and the majority (76.8%) of their husbands were farmers. Two hundred twenty‐six (45.1%) of the households had an average monthly income of 6000 to 12,000 Ethiopian birr (ETB), with a mean and ± SD of income of 7155 ± 2100 ETB. The majority (84.0%) of the study participants have no media exposure, and 70.7% of the women traveled greater than 30 minutes on foot to reach the nearby public health facility (Table 1).

**Table 1:** Sociodemographic characteristics of married women in Shenen Dhugo district, eastern Ethiopia, 2023 (*n* = 501).

Variables	Category	Frequency	Percentage
Age in years	15–24	62	12.4
	25–34	276	55.1
	35–49	163	32.5

Religion	Muslim	372	74.3
	Orthodox	111	22.2
	Protestant	18	3.6

Family size	≤ 5	330	65.9
	> 5	171	34.1

Education level	Unable read and write	51	10.2
	Primary school	136	27.1
	Secondary school	238	47.5
	College and above	76	15.2

Husband education	Unable read and write	31	6.2
	Primary school	48	9.6
	Secondary school	209	41.7
	College and above	213	42.5

Occupation	House wife	436	87.0
	Merchant	45	9.0
	Government employee	20	4.0

Husbands′ occupation	Farmer	385	76.8
	Merchant	72	14.4
	Government employee	44	8.8

Having media exposure	No	421	84.0
	Yes	80	16.0

Average monthly income (in ETB)	> 12,000	77	15.4
	6000–12,000	226	45.1
	< 6000	198	39.5

Distance from nearby health facility	≤ 30 minutes	147	29.3
	30 minutes	354	70.7

Abbreviation: ETB, Ethiopian birr.

### 3.2. Reproductive‐Related Characteristics

The majority (58.7%) of study participants were married at 18 years and above. The majority (61.5%) of them had their first pregnancy before the age of 20 years. The majority (58.5%) of the women had greater than or equal to four gravida, and six in every seven women had greater than or equal to two parities. Almost all (97.0%) had no abortion history, and around 94.2% had no stillbirth. More than half of the women reported pregnancy intention within the next 12 months. Likewise, more than half of the women desired five or more children, whereas about 92.6% of women had fewer than or equal to five alive children. About 61.5% of the women had used at least one type of modern contraceptive before the study period (Table 2).

**Table 2:** Reproductive‐related characteristics of the married women in Shenen Dhugo district, eastern Ethiopia, 2023 (*n* = 501).

Variables	Categories	Frequency	Percentage
Age at first marriage (years)	< 18	207	41.3
	≥ 18	284	58.7
Age at first pregnancy (years)	< 20	308	61.5
	≥ 20	193	38.5
Gravida	< 4	208	41.5
	≥ 4	293	58.5
Para	≥ 2	426	85.0
	< 2	75	15.0
Having abortion history	Yes	15	3.0
	No	486	97.0
Having stillbirth history	Yes	29	5.8
	No	472	94.2
Number of live children	≥ 5	464	92.6
	> 5	37	7.4
Pregnancy intention within next 12 months	Yes	266	53.1
	No	235	46.9
Number of desired children	< 5	240	47.9
	≥ 5	261	52.1
Previous contraceptive use	No	192	38.3
	Yes	309	61.7

### 3.3. Dimensions of Contraceptive Ideation

The contraceptive ideation was assessed using five dimensions. The mean (± SD) of social interaction was 5.4 (± 4.3), with more than half (53.9%) of participants having good social interaction. The mean (± SD) of attitude toward contraceptives was 5.4 (± 3.3), with 39.9% of them having a positive attitude toward contraceptives. The mean (± SD) of perceived self‐efficacy was 4.0 (± 2.3), and half (50.3%) of the participants had high self‐efficacy on contraceptives. Regarding contraceptive awareness, the mean (± SD) was 5.2 (± 1.9), and almost 30% were aware of contraceptives. In addition, the mean (± SD) of perceived norms was 6.4 (± 2.4), and about 54.9% of participants had the perceived right to a contraceptive norm among nonusers of contraceptives (Table 3).

**Table 3:** Contraceptive ideation among married women in Shenen Dhugo district, eastern Ethiopia, 2023.

Dimension of ideation	Categories	Frequency	Percentage
Social interaction	Poor	231	46.1
	Good	270	53.9
Attitude toward contraceptives	Negative	302	60.3
	Positive	199	39.7
Perceived self‐efficacy	Poor	249	49.7
	Good	252	50.3
Awareness about contraceptives	Poor	351	70.1
	Good	150	29.9
Perceived norms	Wrong	226	45.1
	Right	275	54.9

### 3.4. Magnitude of Intention to Use Contraceptives

This study revealed that the magnitude of intention to use modern contraceptives was 48.5% (95% CI: 44.1%, 52.9%) within the next 12 months among nonuser married women in Shenen Dhugo district.

### 3.5. Association of Ideation With Intention to Use Contraceptives

The findings of this study revealed that all five dimensions of ideation were positively associated with intention to use contraceptives among married women. Women with positive or right dimension responses were more likely to intend to use contraceptives than those with negative or wrong. The odds of intention to use modern contraceptives within next 12 months were 4.54, 3.56, 2.04, 3.76, and 2.82 times higher among women who have higher score of social interaction (AOR = 4.54, 95% CI: 2.73, 7.53), attitude (AOR = 3.56, 95% CI: 2.11, 6.00), perceived self‐efficacy (AOR = 2.04, 95% CI: 1.45, 3.79), awareness (AOR = 3.76, 95% CI: 1.94, 7.29), and perceived norms (AOR = 2.82, 95% CI: 1.70, 4.69), respectively, compared with their counterparts. In addition, there is a strong association between education levels, household income, and previous use of contraceptive methods and intention to use contraceptives. The women who had a secondary school education level were three times more likely to intend to use contraceptives (AOR = 3.25, 95% CI: 1.20, 8.80) compared with those who were unable to read and write. The odds of intention to use modern contraceptives among women who have higher income were three times higher (AOR = 3.15, 95% CI: 1.44, 6.90) than those who have lower income. The women who had ever used modern contraceptives were 5.74 times more likely to intend to use contraceptives (AOR = 5.74, 95% CI: 3.27, 10.07) compared with those who had never used (Table 4).

**Table 4:** Association of ideation and other sociodemographic factors with intention to use contraceptives among married women in Shenen Dhugo district, eastern Ethiopia, 2023.

Variables	Responses	Contraceptive intention		COR (95% CI)	*p*	AOR (95% CI)	*p*
		Yes *n* (%)	No *n* (%)				
Education status	Unable read and write	8 (15.7)	43 (84.3)	1		1	
	Primary school	42 (30.9)	94 (69.1)	2.40 (1.04, 5.55)	0.040	1.04 (0.36, 2.98)	0.945
	Secondary school	138 (58.0)	100 (42.0)	7.42 (3.34, 16.46)	< 0.001	3.25 (1.20, 8.80)	0.020
	College and above	55 (72.4)	21 (27.6)	14.08 (5.68, 34.86)	< 0.001	0.97 (0.28, 3.30)	0.959

Monthly income in ETB	> 12,000	40 (51.9)	37 (48.1)	1.53 (0.90, 2.60)	0.115	3.15 (1.44, 6.90)	0.004
	6000–12,000	121 (53.5)	105 (46.5)	1.63 (1.11, 2.40)	0.013	1.59 (0.93, 2.71)	0.091
	< 6000	82 (41.4)	116 (58.6)	1		1	

Distance from nearby health facility	≤ 30 min	68 (46.3)	79 (53.7)	0.88 (0.60, 1.29)	0.157	1.62 (0.92, 2.83)	0.094
	> 30 min	175 (49.4)	179 (50.6)	1		1	

Exposed to media	Yes	61 (76.3)	19 (23.8)	4.22 (2.43, 7.30)	< 0.001	1.38 (0.63, 3.04)	0.421
	No	182 (43.2)	239 (56.8)	1		1	

Family size	≤ 5	178 (53.9)	152 (46.1)	1.91 (1.31, 2.78)	0.001	0.67 (0.38, 1.20)	0.178
	> 5	65 (38.0)	106 (62.0)	1		1	

Ever used contraceptives	Yes	196 (63.4)	113 (36.6)	5.35 (3.58, 8.00)	< 0.001	5.74 (3.27, 10.07)	< 0.001
	No	47 (24.5)	145 (75.5)	1		1	

Social interaction	Poor	64 (27.7)	167 (72.3)	1	< 0.001		< 0.001
	Good	179 (66.3)	91 (33.7)	5.13 (3.50, 7.53)		4.54 (2.73, 7.53)	

Attitude	Negative	110 (36.4)	192 (63.6)	1	< 0.001		< 0.001
	Positive	133 (66.8)	66 (33.2)	3.52 (2.41, 5.13)		3.56 (2.11, 6.00)	

Perceived self‐efficacy	Poor	82 (32.9)	167 (67.1)	1	< 0.001		
	Good	161 (63.9)	91 (36.1)	3.60 (2.49, 5.21)		2.04 (1.45, 3.79)	0.001

Awareness	Poor	128 (36.5)	223 (63.5)	1	< 0.001		
	Good	115 (76.7)	35 (23.3)	5.72 (3.702, 8.86)		3.76 (1.94, 7.29)	< 0.001

Perceived norms	Wrong	71 (31.4)	155 (68.6)	1	< 0.001		
	Right	172 (62.5)	103 (37.5)	3.65 (2.51, 5.29)		2.82 (1.70, 4.69)	< 0.001

Abbreviations: AOR, adjusted odds ratio; COR, crude odds ratio; ETB, Ethiopian birr.

## 4. Discussion

This study assessed the role of ideation on intention to use modern contraceptives within the next 12 months among married women who are currently not using contraceptives. This study revealed the dimensions of ideation, the main determinants of contraceptive use intention in eastern Ethiopia. Dimensions of contraceptive ideation, social interaction, attitude toward contraceptives, perceived self‐efficacy, awareness about contraceptives, and perceived norms were found to be the main predictors of contraceptive use intention. In addition, sociodemographic (secondary school education level and higher income) and reproductive‐related variables (having used previous contraceptives) were the factors improving the women′s intention to use contraceptives.

The finding of this study indicated that the proportion of intention to use modern contraceptives was 48.5% (95% CI: 44.1%, 52.9%) among married women who were nonusers of contraceptives over the next 12 months. The finding of this study was in line with the studies conducted in Ethiopia, 44.1% [28]; Debre Markos, northern Ethiopia, 45.9% [33]; Tigray, northern Ethiopia, 52.1% [34]; Gambela, 50.1% [30]; Mozambique, 44.70% [35]; and Ghana, 52.2% [5]. However, this finding was lower than studies conducted in Aksum, northern Ethiopia, 84.3% [16]; Benishangul‐Gumuz, 74.9% [30]; Malawi, 69.1% [25]; Uganda, 69% [36]; and Bangladesh, 82% [37]. This discrepancy may be due to differences in the reproductive characteristics of the study participants. For example, the comparable study conducted in Aksum, northern Ethiopia, was conducted among women in the postpartum period, whereas our study included all married women in the reproductive age group. The women in the postpartum period may be more likely to intend to use contraceptives than other women.

On the other hand, this study′s finding was higher than the studies conducted overall in Ethiopia, 39.4% [38]; Wellega, western Ethiopia, 18.2% [15]; Wolaita, southern Ethiopia, 38% [39]; Afar, northern Ethiopia, 21.8% [30]; Somali, eastern Ethiopia, 20.1% [30]; Ghana, 31.7% [20]; and Pakistan, 42% [40]. This difference might be attributable to the sociodemographic characteristics of the study participants; the comparable studies were conducted in a pastoralist community [30], where the intention and utilization of modern contraceptives were less likely because of cultural norms, mobility patterns, and limited access to health services. In addition, this variation might be due to methodological differences across the studies. Although our study assessed both short‐acting and long‐acting reversible contraceptives, the comparable studies conducted in Wellega and Wolaita were focused exclusively on long‐acting reversible and permanent contraceptives [15, 39].

In this study, the important role of dimensions of ideation in intention to use contraceptives was identified, indicating that an ideation framework is relevant in developing strategies for increasing the utilization of contraceptives. The findings of this study revealed that a higher contraceptive ideation score was significantly associated with a greater intention to use contraceptives within the next 12 months. This finding was consistent with the studies, suggesting that higher contraceptive ideation is an essential key element to increasing intention to use contraceptives [23, 24, 30, 41].

This study identifies significant dimensions of contraceptive ideation, which are social interaction, attitude toward contraceptives, perceived self‐efficacy, awareness about contraceptives, and perceived norms. Higher contraceptive ideation plays a great role in the enhancement of women′s intention to use contraceptives. It is important to increase the level of intention to use contraceptives among nonusers of contraceptives by promoting discussion about contraceptive use with others [24, 30].

Women who have good social interaction were 4.5 times more likely to have the intention to use contraceptives among nonusers of contraceptives. This implies that women who communicate about contraceptives with their spouses, partners, and peers are more likely to intend to use contraceptives. In addition, spousal communication about contraceptives may strengthen decision‐making autonomy by increasing women′s empowerment and enhancing joint decision‐making. Furthermore, couple discussion on contraceptives can improve couples′ mutual understanding about contraceptives and reduce misconceptions. Overall, husband involvement has played an important role in increasing intention to use contraceptives [42]. Given that, providing practice opportunities for partners on how to communicate about contraceptives is an essential strategy to improve intention to use contraceptives. This study also indicated that self‐efficacy is found to be the main predictor of the intention to use contraceptives. Communication campaigns should focus on enhancing self‐efficacy for contraceptive usage while also addressing common myths and rumors that impede contraceptive utilization.

Women who have a positive attitude toward contraceptives were 3.56 times more likely to have the intention to use contraceptives among nonusers of contraceptives. Women with a positive attitude toward contraceptives are more likely to intend to use contraceptives because attitude is the primary determinant of behavioral intention. In addition, this might be because women with a positive attitude tend to perceive higher benefits and lower misconceptions about contraceptives. In this study, the perceived norms dimension of ideation was found to be a significant predictive variable for the intention to use contraception. This implies that rejection of the myths and rumors about contraceptives in the community increases the level of intention to use contraceptives. It is important to improve the intention to use contraception by eliminating myths and rumors about contraception and using strategically designed messages that provide true information about contraception. In addition, mass media and community conversations that allow people to explore common myths about contraception in their community, while also critically examining their own beliefs about contraception, may be beneficial [24, 30]. In addition to the dimension of ideation, this study identified the important predictors of intention to use contraceptives. The study showed that women who had higher incomes were three times more likely to intend to use contraceptives than those who had lower incomes. This implies that women′s economic status is a significant predictor of contraceptive utilization [26, 43]. It is important to improve women′s socioeconomic status to promote the level of contraceptive intention and utilization. We were surprised that media exposure, which showed a strong association with intention to use contraceptives in bivariable analysis, lost its association in the multivariable model. This suggests that media exposure has no direct association with intention, but acts as a mediator through ideation dimensions.

Women who had a secondary school education level were more likely to intend to use contraceptives compared with those unable to read and write. This might be due to women who never attend formal education having less knowledge and awareness about contraceptives. In addition, women with no education may have limited access to accurate information about contraceptives, leading to fear, misconceptions, and hesitancy to use [14]. Furthermore, a lower education level can contribute to lower self‐esteem and limited belief in the ability to make informed decisions regarding reproductive health services and can discourage contraceptive use [44]. In contrast, college and above education did not show a significant association with intention to use contraceptives. We were surprised by this finding, as higher education is assumed to enhance reproductive health awareness and self‐efficacy. This might be due to the small sample size within the college and above category, which may have limited the statistical power to detect an association. In addition, this possibility might be due to the majority of women with higher education being excluded from the study, as they were using contraceptives because this study enrolled only nonusers.

This study indicated that the women who had ever used previous contraceptives were four times more likely to intend to use contraceptives, compared with those who had never used. This finding was supported by the studies conducted in Liberia, which indicated that women who have used contraceptives were more likely to intend to use contraceptives [38]. This may be due to positive experiences with previous contraceptives improving the women′s perception, and it can enhance the women′s perceived control over their use [45]. Given that, having successful contraceptive use in the previous period may improve women′s confidence in contraceptive accessibility and effective management, which reinforces their intention to use contraceptives again [38].

The limitation of this study is that the cross‐sectional study design limited its ability to identify causal effects between ideation dimensions and the intention to use contraceptives. Limiting the study to currently married nonusers restricts generalizability to unmarried individuals and contraceptive users. Using the mean as the cutoff for dichotomizing ideation scores introduces a sample‐specific threshold that is not externally validated, reducing comparability across studies. In addition, recruiting health extension workers from the same community as the data collectors may have contributed to social desirability bias beyond what interviewing in a separate room can address. Furthermore, the single‐district study setting may limit generalizability to other parts of eastern Ethiopia.

## 5. Conclusion

This study shows that nearly half of married women who are nonusers of contraceptives intended to use contraceptives in Shenen Dhugo, eastern Ethiopia. This study provides evidence about the role of ideation in intention to use contraceptives and identifies the importance of ideation in helping to promote women′s intention to use contraceptives. Therefore, developing interventions that improve women′s contraceptive ideation is a key innovative strategy to enhance contraceptive utilization. Encouraging women and couples to discuss and determine a preferred family size is essential to promote contraceptive self‐efficacy. In addition, providing behavioral change health education on contraceptives at every entry point in the health system while addressing existing myths and rumors regarding contraceptive side effects is necessary to strengthen intention and utilization of contraceptives. Furthermore, improving communication and awareness about contraceptives through promoting discussion about contraceptive use with significant others and providing effective health education about contraceptives is important to enhance women′s intention to use contraceptives.

**Nomenclature**
AOR: adjusted odds ratio
FP: family planning
LIMIC: lower‐middle‐income countries
SSA: sub‐Saharan Africa
WHO: World Health Organization

## Author Contributions

A.B., B.G., A.A.U., H.A.A., and M.G. participated in the conception of the idea, development, and amendment of the proposal, data collection, analysis, and writing up the results. A.B., B.G., A.A.U., H.A.A., and M.G. analyzed the data. A.A.U. drafted the manuscript, with continuous input from A.B., B.G., H.A.A., and M.G., who reviewed the manuscript for intellectual content.

## Funding

No funding was received for this manuscript.

## Disclosure

All authors read and approved the final manuscript.

## Conflicts of Interest

The authors declare no conflicts of interest.

## Supporting information


**Supporting Information** Additional supporting information can be found online in the Supporting Information section. Data S1: Dimensions of ideation assessment items on intention to use contraceptives among married women in Shenen Dhugo district, eastern Ethiopia, 2023.

## Data Availability

The data that support the findings of this study are available from the corresponding author upon reasonable request.
