# Supplementary material for: The Role of Ideation on Intention to Use Contraceptives Among Married Women in Shenen Dhugo District, Eastern Ethiopia
Source: Biomed Res Int. 2026 Sep 16;2026:9361411. doi: 10.1155/bmri/9361411 (PMC13579532; doi:10.1155/bmri/9361411)
Supplement: Supplementary file 1 [file BMRI-2026-9361411-s001.pdf]

Dimensions of Ideation Assessment Items on Intention to Use Contraceptives among married women in Shenen Dhugo district, eastern Ethiopia, 2023

Items used to assess the Dimension of Ideation of intention to use contraceptives		Responses				
**Social interaction: 12 items Likert scales**		**0=Never, 1= Rarely, 2=Sometimes, 3=Often, 4=Very Often**				
		**0**	**1**	**2**	**3**	**4**
	How often discussed FP with mother	☐	☐	☐	☐	☐
	How often discussed FP with mother-in-law	☐	☐	☐	☐	☐
	How often discussed FP with aunts	☐	☐	☐	☐	☐
	How often discussed FP with sister	☐	☐	☐	☐	☐
	How often discussed FP with sister-in-law	☐	☐	☐	☐	☐
	How often discussed FP with father	☐	☐	☐	☐	☐
	How often supported contraceptive use by the mother	☐	☐	☐	☐	☐
	How often supported contraceptive use by the mother-in-law	☐	☐	☐	☐	☐
	How often supported contraceptive use by sister-in-law	☐	☐	☐	☐	☐
	How often supported contraceptive use by the father	☐	☐	☐	☐	☐
	How often supported contraceptive use by the father-in-law	☐	☐	☐	☐	☐
	How often supported contraceptive use by religious leaders	☐	☐	☐	☐	☐
**Attitude toward contraceptives: 15 items of Likert scales**		**0=Strongly Disagree, 1=Disagree, 2=Neutral, 3=Agree, 4=Strongly Agree**				
		**0**	**1**	**2**	**3**	**4**
	Women should have the right to decide on the number of children	☐	☐	☐	☐	☐
	Women should say her feelings on the number of children	☐	☐	☐	☐	☐
	Women should discuss about contraceptives with partner	☐	☐	☐	☐	☐
	Women should discuss about contraceptives with family/relatives	☐	☐	☐	☐	☐
	Women should discuss about contraceptives with friends	☐	☐	☐	☐	☐
	Women should discuss about contraceptives with neighbors	☐	☐	☐	☐	☐
	Women should discuss about contraceptives with colleagues	☐	☐	☐	☐	☐
	Use of contraceptives has benefits for women	☐	☐	☐	☐	☐

	Partners have positive attitude toward contraceptive use	☐	☐	☐	☐	☐
	Religion leaders have positive attitude toward contraceptive	☐	☐	☐	☐	☐
	Large family size is respected by husband	☐	☐	☐	☐	☐
	Large family size is respected by community	☐	☐	☐	☐	☐
	Large family size is favorable by religious	☐	☐	☐	☐	☐
	Large family size can negatively affect the economy of the country	☐	☐	☐	☐	☐
	Large family size can affect the health of the mothers	☐	☐	☐	☐	☐
**Perceived self-efficacy: 7 items Likert Scales**		**0=Strongly Disagree, 1=Disagree, 2=Neutral, 3=Agree, 4=Strongly Agree**				
		**0**	**1**	**2**	**3**	**4**
	Perceived self-efficacy for starting a conversation with partner about FP	☐	☐	☐	☐	☐
	Perceived self-efficacy for convincing partner that they should use a FP method	☐	☐	☐	☐	☐
	Perceived self-efficacy for obtaining a FP method if decided to use one	☐	☐	☐	☐	☐
	Perceived self-efficacy for using a FP method even if partner doesn't want	☐	☐	☐	☐	☐
	Perceived self-efficacy for using a FP method even if no friend or neighbor uses	☐	☐	☐	☐	☐
	Perceived self-efficacy for using a FP method even if religious leader disagrees	☐	☐	☐	☐	☐
	Perceived self-efficacy for getting to a place where contraceptives are provided	☐	☐	☐	☐	☐
**Awareness about contraceptives: 12 dichotomized items**						
	Aware of female sterilization	**0= No**	**1= Yes**			
	Aware of male sterilization	**0= No**	**1= Yes**			
	Aware of IUD	**0= No**	**1= Yes**			
	Aware of injectables	**0= No**	**1= Yes**			
	Aware of implants	**0= No**	**1= Yes**			
	Aware of pills	**0= No**	**1= Yes**			

	Aware of male condom	**0= No 1= Yes**				
	Aware of female condom	**0= No 1= Yes**				
	Aware of lactational method	**0= No 1= Yes**				
	Aware of rhythm method	**0= No 1= Yes**				
	Aware of withdrawal failure	**0= No 1= Yes**				
	Aware of emergency contraceptive	**0= No 1= Yes**				
**Perceived norms: 12 items Likert-scale**		**0=Strongly Disagree, 1=Disagree, 2=Neutral, 3=Agree, 4=Strongly Agree**				
		**0**	**1**	**2**	**3**	**4**
	Perceived sterility by injection of contraceptive	☐	☐	☐	☐	☐
	Perceived health problem by contraceptive use	☐	☐	☐	☐	☐
	Perceived the uterus is harmed by Contraceptive use	☐	☐	☐	☐	☐
	Perceived sexual urge reduced by contraceptive use	☐	☐	☐	☐	☐
	Perceived cancer brought by contraceptive use	☐	☐	☐	☐	☐
	Perceived deformed baby brought by contraceptive use	☐	☐	☐	☐	☐
	Perceived dangerous of contraceptive use if no balanced diet	☐	☐	☐	☐	☐
	Perceived dangerous of contraceptive use to women's health	☐	☐	☐	☐	☐
	Perceived that family planning use make promiscuous	☐	☐	☐	☐	☐
	Perceived that women cannot use contraceptives if they cook	☐	☐	☐	☐	☐
	Perceived that women did not get enough nutrition should not use contraceptives	☐	☐	☐	☐	☐
	Perceived disappearance of intrauterine device by itself in the body	☐	☐	☐	☐	☐
